# *Alisma orientalis* Beverage Treats Atherosclerosis by Regulating Gut Microbiota in ApoE^-/-^ Mice

**DOI:** 10.3389/fphar.2020.570555

**Published:** 2020-09-25

**Authors:** Boran Zhu, Yi Zhai, Mengjiao Ji, Yanan Wei, Jiafei Wu, Wenda Xue, Wei wei Tao, Haoxin Wu

**Affiliations:** School of Chinese Medicine, Nanjing University of Chinese Medicine, Nanjing, China

**Keywords:** atherosclerosis, traditional Chinese medicine, *Alisma orientalis* beverage, trimethylamine N-oxide, gut microbiota, herb formula

## Abstract

**Background:**

*Alisma orientalis* beverage (AOB) is a Chinese traditional medicine formulated with a diversity of medicinal plants and used for treating metabolic syndrome and atherosclerosis (AS) since time ago. Given the current limited biological research on AOB, the mechanism by which AOB treats AS is unknown. This study investigats the role of AOB-induced gut microbiota regulation in the expansion of AS.

**Methods:**

We established an AS model in male apolipoprotein E-deficient (ApoE^−/−^) mice that are fed with a high-fat diet (HFD), treated with numerous interventions, and evaluated the inflammatory cytokines and serum biochemical indices. The root of the aorta was stained with oil red O, and the proportion of the lesion area was quantified. Trimethylamine N-oxide (TMAO) and trimethylamine (TMA) levels in serum were evaluated through liquid chromatography with mass spectrometry. Flavin−containing monooxygenase 3 (FMO3) liver protein expression was assessed by Western blotting. 16S rDNA sequencing technique was adopted to establish the changes in the microbiota structure.

**Results:**

After 8 weeks of HFD feeding, an inflammatory cytokine, and AS development expression were significantly decreased in mice treated with AOB; the same parameters in the mice treated with the antibiotics cocktail did not change. In the gut microbiota study, mice treated with AOB had a markedly different gut microbiota than the HFD-fed mice. Additionally, AOB also decreased serum TMAO and hepatic FMO3 expression.

**Conclusion:**

The antiatherosclerotic effects of AOB were found associated with changes in the content of gut microbiota and a reduction in TMAO, a gut microbiota metabolite, suggesting that AOB has potential therapeutic value in the treatment of AS.

## Introduction

Despite considerable advances in its prevention, diagnosis and treatment, atherosclerosis (AS) is still a major reason for mortality around the world (Libby et al., 2011; Bentzon et al., 2014). AS is closely related to the high-fat Western diet, which is related with chronic inflammation of the vessel wall that can block blood flow (Kiouptsi et al., 2019). The microbiota of the gut is involved in the process of AS as a result of poor eating habits, including the excessive use of dietary choline, red meat and fat (Wang et al., 2015; Koeth et al., 2019; Wang et al., 2019).

Dietary molecules are transformed into trimethylamine (TMA) through the action of gut microbiota, and TMA is then transformed into trimethylamine N-oxide (TMAO) by hepatic flavin monooxygenase (FMO). FMO3 is a major member of the FMO family, and inhibition of FMO3 can significantly decrease TMAO levels and AS (Shih et al., 2019). TMAO as a gut microbiota-derived metabolite, which is also related to the Western diets (Barrea et al., 2019a). It may promote the development of obesity (Barrea et al., 2019b) and NAFLD (Barrea et al., 2018). In recent years, TMAO is considered to be a prognostic marker for the development of AS into cardiovascular disease (CVD) (Wang et al., 2011; Koeth et al., 2013). A latest systematic review demonstrated a dose-dependent positive correlation among TMAO levels and increased CVD risk and mortality (Schiattarella et al., 2017). Numerous mechanisms have been suggested, including the high scavenger receptors expression of on macrophages mediated by TMAO, promoting foam cell formation; bile acid, sterol, and cholesterol metabolism; changes in sterol transporter; and the activation of proinflammatory mechanisms (Nam, 2019).

Chinese Traditional medicine has a history of 2,000 years and has received massive biomedical efficacy. Plenty of traditional Chinese medications, such as Tongxinluo and Xuezhikang, were seen as an alternative and complementary strategy to primary and secondary CVD prevention (Hao et al., 2015; Hao et al., 2017). *Alisma orientalis* beverage (AOB), a traditional formula in Chinese medicine documented in the book of Hunagdineijing, is the earliest Chinese medicine. The main ingredients in AOB are the decoction of *Alisma plantago-aquatica subsp. orientale* (Sam.) Sam. (zexie), *Atractylodes macrocephala* Koidz. (baizhu), and *Pyrola calliantha* Andres (luxiancao), which interferes with lipid metabolism and reduces atherosclerosis based on the Chinese Pharmacopeia (2015 Edition). Currents report demonstrated that *Alisma plantago-aquatica subsp. orientale* (Sam.) Sam. has antisteatosis effects that involve lipogenesis, antilipoapoptosis, and anti-inflammation, and it also contributes to a lipid-lowering effect in individuals with high-fat diet- hyperlipidemia induced (Jeong et al., 2016; Li et al., 2019). Chinese medicines containing *Atractylodes macrocephala* Koidz. reduced the serum of total cholesterol (TC) and inflammatory cytokines and upgraded microbiota of the gut (Zhang et al., 2018; Zhao et al., 2018).

## Material and Methods

### Drugs and Diet

AOB composed of three species of Chinese traditional herbs, and its composition is indicated in **Table 1**. All herbs were obtained from Nanjing GuoYi Clinical, Medicinal Material Department (Nanjing, China). Voucher specimens of *Alisma plantago-aquatica subsp. orientale* (Sam.) Sam. (voucher number: NZY-ZHU-2019001), *Atractylodes macrocephala* Koidz. (voucher number: NZY-ZHU-2019002), and *Pyrola calliantha* Andres (voucher number: NZY-ZHU-2019003) had been deposited in the Herbarium of, Nanjing University of Chinese medicine of Chinese Traditional Medicine. Medicinal herbs have been blended and extraction was carried out at 95°C with distilled water used in 10 volumes of the mass of the blend (v/m) while stirring for 1 h. This was followed by the centrifugation of the extract, thus concentrating it to 1 and 0.5 g/mL (net content). The final stock solution of AOB stocking was stored at -20°C. Before administration, AOB was filtered using sterilized 0.22-μm syringe filters. The quality of different batches of AOB was tested through Waters 2695 Alliance HPLC system (Waters Corp, Milford, MA, U.S.A.) method. Briefly, HPLC was performed AOB and reference standards solutions. The mobile phases chosen were acetonitrile (A) and 0.1% aqueous formic acid mixture (B). The mobile gradient phase was as follows: 5 to 50% A in 0 to 10 min, 50 to 100% A in 10 to 40 min, 100% A in 40 to 43 min to elute the specimens. The temperature of the oven was maintained at 30°C while the flow rate was 1 mL/min. The chromatographic column of Apollo C18 (250 mm/4.6, 5 μm, Apollo, Milford, MA, U.S.A.) was used. The supplemental materials give comprehensive chemical information about AOB. The cocktail of antibiotics consisted of four antibiotics including vancomycin (50 mg/kg, Amresco Inc.), metronidazole (100 mg/kg, MP Biomedicals), ampicillin (100 mg/kg, Amresco Inc.), and neomycin (100 mg/kg, Amresco Inc.) (Zarrinpar et al., 2018). Atorvastatin (1.3 mg/kg, Pfizer Inc) was used as a positive control against AS. The HFD contains 77.75% regular chow, 20% lard, 1.25% cholesterol, and 1% choline (no. XS-2019-11-12-04204 purchased from Nanjing Junke Biotechnology Co, Ltd., China).

**Table 1:** The composition of AOB.

Chinese name	Latin name	Family	Part used	Ratio	Batch number
Ze xie	*Alisma plantago-aquatica subsp. orientale* (Sam.) Sam.	Alismataceae Vent.	Corms	2	180903
Bai zhu	*Atractylodes macrocephala* Koidz.	Asteraceae Bercht. and J.Presl	Rhizome	2	180903
Lu xian cao	*Pyrola calliantha* Andres	Ericaceae Durande	Whole plant	1	180903

### Animals and Groups

Male apolipoprotein E-deficient (ApoE^−/−^) mice (8 ± 1 weeks old, 25 ± 5 g) were acquired from Changzhou Cavns Laboratory Animal Co., Ltd. All the mice were held at a temperature of 23 ± 2°C and a light/dark period of 12 h under normal conditions. Excluding the experimental time outside the tank, unlimited access to water and food was provided to the animals. Four to six mice were kept in one cage. The experiments were conducted from 09:00 am to 3:00 pm using all possible efforts. All the procedures were carried by following the Animal Care and Use Committee (Key Laboratory of Integrative Medicine for Brain Diseases, Nanjing University of Chinese Medicine) Institutional Guidelines. All animal experiments were performed in compliance with the Guide to the Care and Use of Laboratory Animals authorized by the Committee for Institutional Animal Care and Use at the University of Chinese Medicine in Nanjing.

One week following the adaptive breeding, mice were distributed into 7 groups: the normal control diet group (NC, n = 6), in which the mice were fed for 8 weeks with a normal diet; the high-fat diet group (HFD, n = 6), in which the mice were fed with a high-fat diet for 8 weeks; the high-dose AOB gavage group (AOBH, n = 6), in which the mice were fed with a high-fat diet and intragastrically injected AOB (6.5 g/kg^-1^ d^-1^) for 8 weeks; the low-dose AOB gavage group (AOBL, n = 6), in which the mice were fed with a high-fat diet and intragastrically injected 3.25 g/kg^-1^ d^-1^ AOB for 8 weeks; the antibiotics cocktail gavage group (Abs, n = 6), in which the mice were fed with a high-fat diet and intragastrically administered an antibiotics cocktail (ampicillin, 100 mg/kg^-1^ d^-1^; neomycin, 100 mg/kg^-1^ d^-1^) metronidazole (100 mg/kg^-1^ d^-11^), vancomycin (50 mg/kg^-1^ d^-1^) for 8 weeks; the antibiotics cocktail combined with high-dose AOB gavage group (AbsA, n = 6), in which the mice were fed with a high-fat diet and intragastrically administered an antibiotics cocktail as previous described and AOB (6.5 g/kg^-1^ d^-1^) for 8 weeks; and the atorvastatin gavage group (Ato, n = 6), in which the mice were fed with a high-fat diet and intragastrically injected atorvastatin (1.3 mg/kg^-1^ d^-1^) for 8 weeks.

### Lipid Analysis

All mice were weighed and anesthetized with 1% pentobarbital sodium after eight weeks of feeding (30 mg/kg, intraperitoneally). Blood extracted from cardiac puncture was further centrifuged for 10 at 3000 rpm min while maintain the temperature at 4°C. A Chemray 240 full-automatic biochemical analyzer (Wuhan Servicebio Technology, Co., Ltd, China) tested high-density lipoprotein cholesterol (HDL-C) and triglycerides (TG), the serum levels of TC and low-density lipoprotein cholesterol (LDL-C). All experiments were conducted as per instructions from the manufacturer.

### Aortic Lesion Analysis

After blood collection, the mice were perfused with PBS (0.1 mol/L) transcardially. Hearts were fixed with optimal cutting temperature compound, and then the aortic roots were sliced into consecutive sections (10 µm) from the aortic sinus to the aortic arch for histological examination lesions in the aortic sinus of atherosclerotic. Lesion part measurement was based on oil red O staining by taking the mean value of six sections, and images were evaluated by employing the software of Image Pro Plus 6.0 (Image analysis software, Media Cybernetics, Rockville, MD, USA).

### Multiple Cytokine Measurements

The serum systemic inflammatory cytokine levels were evaluated through Mouse Magnetic Luminex Screening Assay 6 PLEX (96-plex, R&D Systems, Minneapolis, MN, USA) as per the instruction of the manufacturer.

### TMA and TMAO Analysis

The samples were tested on a water UHPLC system (Vanquish, Thermo, Waltham, MA, U.S.A.) coupled with mass spectrometry (TSQ Altis, Thermo, Waltham, MA, U.S.A.). Waters Mildford, MA, U.S.A.) and Waters ACQUITY UPLC HSS T3 (100** mm** × 2.1** mm**, 1.8 μm were used to isolate the specimens, and the column was held at 30°C. Mobile phase A contained of 0.1% formic acid in H_2_O, and 0.1% acetonitrile in water was in mobile phase B. MS quadrupole and ion source temperatures (electron impact) were taken as 100 and 650°C, respectively. The calculations were conducted for TMA of range of m/z 60–44, and for TMAO m/z 76-58. Detailed procedures are set out in the Additional Content.

### Cecal Content DNA Extraction and 16S rRNA High-Throughput Sequencing

Microbial DNA was collected from specimens of mouse cecal material using E.Z.N.A. ^®^ soil DNA kit (Omega Biotek, Norcross, GA, U.S.) as per instructions provided by the manufacturer. A NanoDrop 2000 UV-vis spectrophotometer (Thermo Science, Wilmington, USA) was employed to assess the DNA purification and concentration. Furthermore, 1% agarose gel electrophoresis was employed for assessing the DNA quality. A thermocycler polymerase chain reaction (PCR) system (GeneAmp 9700, ABI, USA) was employed for the amplification of the V3-V4 hypervariable parts of the bacterial 16S rRNA gene *via* primers 338F (5’- ACTCCTACGGGAGGCAGCAG-3’) and 806R (5’-G GGACTACHVGGGTWTCTAAT-3’). The PCRs were performed adopting the program as a denaturation for 3 min at 95°C, 27 cycles of the 30 s at 95°C, and 45 s at 72°C for elongation, 30 s at 55°C for annealing, and a final extension at 72°C for 10 min. Triplicate PCRs were conducted in a solution of 20 μL containing 4 μL of 5 FastPfu buffer, 0.8 μL of each primer (5 μM), 2 μL of 2.5 mM dNTPs, 10 ng of DNA template, and 0.4 μL of FastPfu polymerase. The subsequent PCR products were isolated from a 2% agarose gel, and their further purification was achieved with AxyPrep DNA Gel Extraction Kit (Axygen Biosciences, Union City, CA, USA) and measured as per the manufacturer’s protocol using QuantiFluorTM-ST (Promega, USA). Extracted amplicons were clustered on an Illumina MiSeq platform (Illumina, San Diego, USA) in equimolar quantities and paired-end synthesized in accordance with the standard guidelines of Majorbio Bio-Pharm Technology Co. Shanghai (China) Ltd.

### Bioinformatics Analysis

Quality-filtration of raw fastq files was achieved *via* Trimmomatic followed by their merging through FLASH employing the following criteria: (i) the reads were shortened at any site for obtaining an overall quality score of less than 20 over a sliding window of 50 bp. (ii) Sequences having overlap exceeding 10 bp were merged with a mismatch having their bp not more than 2. (iii) Separation of sequences of individual samples was carried out according to barcode technology (exactly matching) and primers (allowing two nucleotide mismatches), and reads having unclear base was omitted. Operational taxonomic units (OTUs) were grouped with a similarity cut-off of 97% utilizing UPARSE (version 7.1; http://drive5.com/uparse/) with a new “greedy” algorithm, which concurrently conducts chimaera sorting and OTU clustering. The RDP supervised learning algorithm (http://rdp.cme.msu.edu/) was used for evaluating the taxonomy of individuals 16S rRNA gene sequence against all the Silva (SSU123) 16S rRNA database that used a confidence threshold of 70%.

### Western Blotting

Livers were obtained, and the samples of proteins for Western blotting (WB) were extracted. The primary antibodies being used were monoclonal rabbit antibody FMO3 (1:5,000) and monoclonal rabbit antibody GAPDH (1:10,000) (both Abcam Inc., Cambridge, MA, USA). The secondary antibody IgG(H+L) used was covalently linked with horseradish peroxidase (1:2,000) (from Cell Signal Inc., CA, USA). An ECL solvent was used for the visualization of immunoreactivity. Visualization of blots was carried out with Chemiluminescent Substrate SuperSignal West Pico (Thermo Fisher Science Inc.) and was displayed as density relative to GAPDH. Experiments were carried out in triplicates.

### Statistical Analysis

All data is shown as means ± SDs. One-way ANOVA was used with the honestly important difference from Tukey or the post-hoc test from Dunnett. For all statistical tests, SPSS v.21 was used, and two-tailed p values below 0.05 were considered statistically important.

## Results

### Effect of AOB on the Ratio of Adipose Tissue on the Body Weight and Serum Lipid Levels

**Figure 1:**
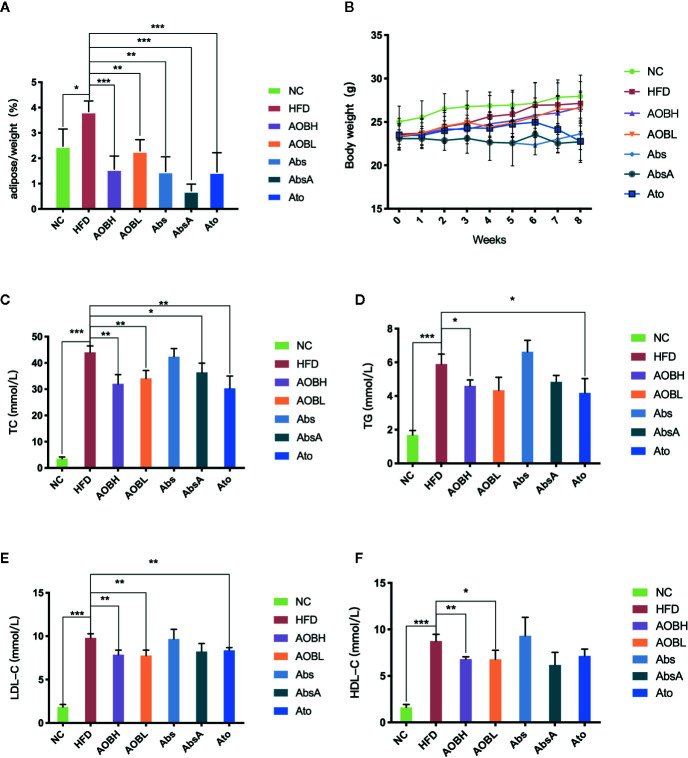
AOB decreased the amount of tissue and lipid content in adipose. **(A)** The ratio of adipose body tissue mass at the ends of the week. **(B)** Growth curves given the average body weight of the seven groups calculated per week. **(C)** TC, **(D)** TG, **(E)** LDL-C, and **(F)** HDL-C levels at 8 weeks in serum samples of the seven groups of mice. Values are shown as the ± standard mean deviation. ANOVA calculated disparities. Compared to the HFD community, *p < 0.05, **p < 0.01, ***p < 0.001.

In order to determine the basic situation of AOB intervention. As seen in **Figures 1A, B**, compared to the HFD group, the weight change in eight weeks was not obvious, but the adipose tissue relative to body weight of each group was reduced at week eight. Among them, HFD group (3.84 ± 0.41%) (p < 0.01) is much higher than the other groups. Regarding blood lipid levels, compared to the NC group, the HFD group shows significantly high amount of serum TC (NC: 3.56 ± 0.62 mmol/l, HFD: 44.07 ± 2.39 mmol/l), TG (NC: 1.69 ± 0.26 mmol/l, HFD: 5.89 ± 0.59 mmol/l), LDL-C (NC: 1.84 ± 0.29 mmol/l, HFD: 9.81 ± 0.47 mmol/l) and HDL-C (NC: 1.62 ± 0.31 mmol/l, HFD: 8.75 ± 0.72 mmol/l) (p < 0.001) (**Figures 1C–F**). After AOB or atorvastatin treatment, the serum levels of TC (AOBH: 32.05 ± 3.47 mmol/l, Ato: 30.31 ± 4.67 mmol/l), TG (AOBH: 4.60 ± 0.36 mmol/l, Ato: 4.18 ± 0.85 mmol/l), and LDL-C (AOBH: 7.87 ± 0.53 mmol/l, Ato: 8.36 ± 0.32 mmol/l) were significantly decreased (p < 0.05) (**Figures 1C–F**). The serum lipid biochemical levels of the Abs group did not change significantly, and the AbsA group, which was treated as the Abs group combined with AOB intervention, had significantly decreased TC (AbsA: 36.39 ± 3.51 mmol/l) levels (p < 0.05) (**Figure 1C**).

### Attenuation of HFD-Induced Atherosclerosis by AOB Treatment

**Figure 2:**
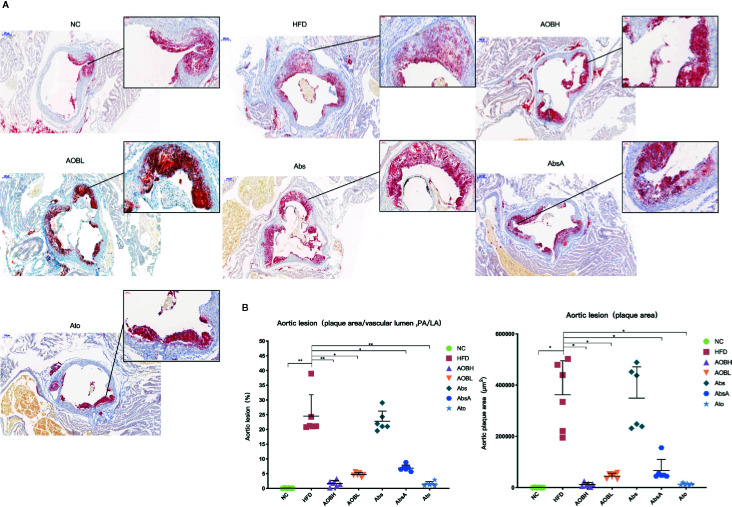
AOB impacts on atherosclerotic lesions. **(A)** Oil red O staining of plaques in the NC, HFD, AOBH, AOBL, Abs, Abs, and Ato aortic root cross-sections. **(B)** Quantitative examination of the percentage of plaque area compared to the vascular lumen area and plaque region; ANOVA calculated disparities. Compared to the HFD community, *p < 0.05, **p < 0.01.

We evaluated the therapeutic impact of AOB on plaque development by histological examination of the cross-sectional lesions of the aortic roots. At the same time, using the amplified image of clogged areas, we can observe that the lipid and foam cells in the plaque stained by red oil O are more dense than other groups (**Figure 2A**). Severe vascular clogging was clearly observed in the HFD (361854.66 ± 132598.21 μm^2^) and Abs groups (349267.83 ± 121761.48 μm^2^), whereas the aortas of the other treatment groups were less clogged (p < 0.05) (**Figure 2B**). As all of the roots of aorta cross-sections were not of the same size or diameter, we analyzed the percentage of plaque area relative to the vascular lumen area. In the HFD group, the percentage was 24.57 ± 7.18, which was not significantly (p > 0.05) different from the average plaque area of the Abs group (22.82 ± 3.44%) (**Figure 2B**). Moreover, the percentage of plaque area to the vascular lumen area was significantly (p < 0.01) reduced in the aortic sections of the AOBH (1.64 ± 1.05%) and Abs (1.50 ± 0.77%) groups. The percentages in the AOBL (4.67 ± 0.65%) and AbsA (6.89 ± 1.05%) groups also decreased (p < 0.05).

### Effects of AOB on Serum Systemic Inflammatory Cytokine Levels

**Figure 3:**
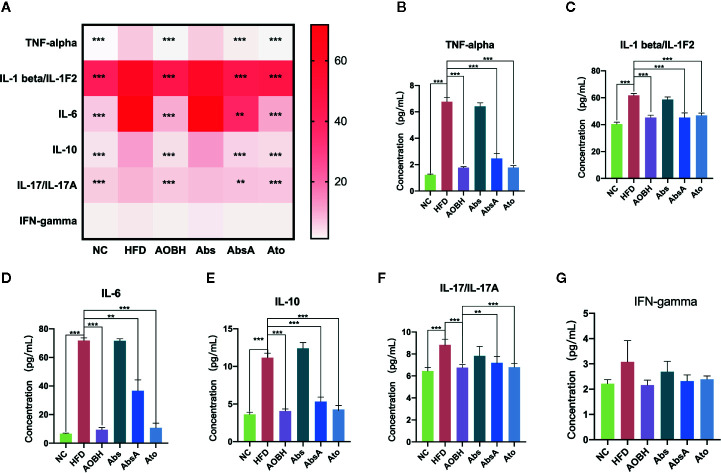
AOB effects on serum cytokine levels and chemokine levels. Quantified serum cytokine levels *via* Mouse Magnetic Luminex Screening Assay 6 PLEX (6 specimens per group). **(A)** 6 cytokine and chemical level heat map. **(B)** TNF-alpha. **(C)** IL-1 beta/IL-1F2. **(D)** IL-6. **(E)** IL-10. **(F)** IL-17/IL-17A. **(G)** IFN-gamma. Values are represented as the ± standard mean deviation. ANOVA calculated disparities. Compared to the HFD community, **p < 0.01, ***p < 0.001.

To determine the AOB systemic effects on decreasing the chronic inflammatory response, multicytokine examination was carried to check the levels of major serum inflammatory cytokines associated with arteriosclerosis. As seen in **Figure 3A**, ApoE^−/−^ mice consuming a HFD had markedly greater contents of interleukin-10 (IL-10), tumor necrosis factor interleukin-6 (IL-16), alpha (TNF-α), interleukin-1-beta (IL-1β), and interleukin-17 (IL-17) than the NC group (p < 0.001). These serum systemic inflammatory cytokine levels did not decrease when antibiotics were given, but they decreased significantly in the AOB (p < 0.001) and atorvastatin-treated groups (p < 0.001). Compared to the HFD group, the most obvious decline in these inflammatory factors by the AOB group are TNF-alpha (HFD: 6.77 ± 0.34 pg/ml, AOBH: 1.77 ± 0.08 pg/ml), IL-1β (HFD: 61.79 ± 1.38 pg/ml, AOBH: 45.27 ± 1.75 pg/ml), IL-16 (HFD: 71.85 ± 1.86 pg/ml, AOBH: 9.45 ± 1.45 pg/ml), IL-10(HFD: 11.17 ± 0.58 pg/ml, AOBH: 4.07 ± 0.28 pg/ml), IL-17 (8.84 ± 0.51 pg/ml, AOBH: 6.76 ± 0.28 pg/ml) (**Figures 3B–F**). IFN-gamma only has a downward trend in the AOB group (HFD: 3.08 ± 0.84, AOBH: 2.16 ± 0.19) (p < 0.1) (**Figures 3A, G**), but this trend does not constitute statistical significance.

### Decreased Hepatic FMO3 Expression and Serum TMAO Levels After AOB Treatment

**Figure 4:**
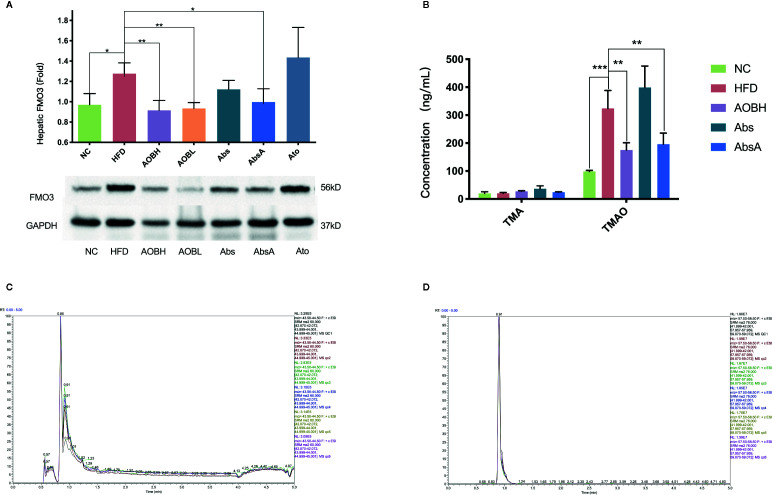
AOB influences hepatic FMO3 thus decreasing the TMA and TMAO serum levels in apoE-/- mice. **(A)** Western blot quantified the FMO3 expression levels in mice. **(B)** Serum concentrations of TMA and TMAO. **(C)** UHPLC/MS/MS assessed serum TMA **(D)** and serum TMAO levels. Values are shown as the ± standard mean deviation. ANOVA calculated disparities. Compared to the HFD community, ***p < 0.05, **p < 0.01, ***p < 0.001.

Serum TMAO is primarily formed by the transformation of hepatic FMO3 from TMA into the liver (Shih et al., 2019). So, we examined if AOB modifies the FMO3 liver expression. we noticed no significant differences in levels of FMO3 in HFD, Abs, and Ato groups, but the FMO3 level in the AOB treatment group (HFD: 1.28 ± 0.11Fold, AOBH: 0.92 ± 0.09Fold, AOBL: 0.93 ± 0.06Fold) (p < 0.01) decreased significantly, and the FMO3 level was also reduced after antibiotics intervention combined with AOB treatment(AbsA: 0.99 ± 0.13Fold) (p < 0.05) (**Figure 4A**). We then studied AOB’s ability to minimize *in vivo* TMA and TMAO. Analysis indicated that AOB patients undergoing decreased the accumulation of TMAO (HFD: 324.09 ± 64.21 ng**/**ml; AOBH: 175.18 ± 25.84 ng**/**ml) in the liver (p < 0.01) (**Figures 4B–D**). This finding indicates that AOB impacts the gut microbiota while regulating FMO3 expression in the liver and reducing the TMAO concentration *in vivo*.

### Role of AOB-Mediated Microbiota Diversity Changes in the Development of Atherosclerosis

**Figure 5:**
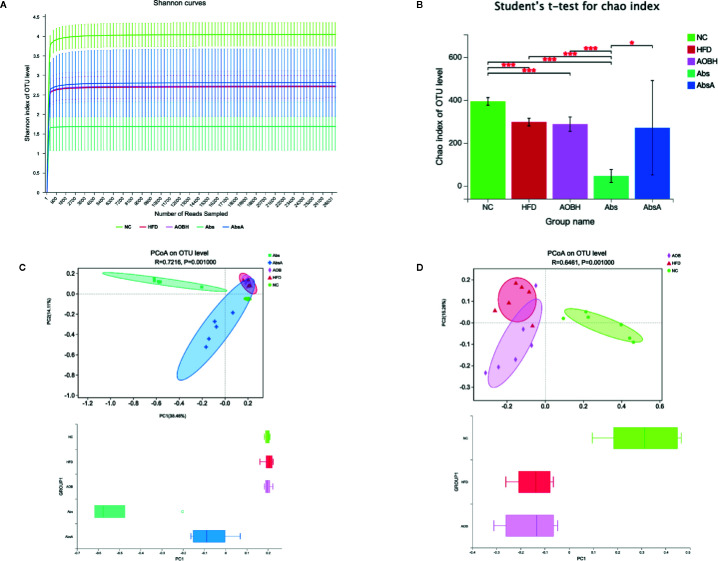
Analyzing the diversity of the gut microbiota among different groups. **(A)** Shannon rarefaction curve in individual groups. The abscissa shows the number of sequencing tags to be retrieved randomly, and ordinates reflect the estimated value of the Sobs index determined when a specific number of tags are retrieved. **(B)** Alpha diversity index (Chao estimator) analysis for each group. Student’s t-test was employed for determination of discrepancies. *p < 0.05, ***p < 0.001 compared to group. **(C)** Principal coordinate analysis (PCoA) for unweighted UniFrac distance metrics among NC, HFD, AOBH, Abs, and AbsA groups in gut microbiota populations. **(D)** PCoA for unweighted UniFrac distance metric in the gut microbiota communities among the NC, HFD, and AOBH groups.

To analyze the effect of AOB treatment on the composition of gut microbiota, we sequenced 1437216 clean reads from 30 samples. The curve within each sample appeared to be flat in the Shannon rarefaction map (**Figure 5A**), indicating that the range of sequencing was able to determine the biodiversity throughout the specimens. The ordinate of the curve of the Abs group was significantly lower than that of the other groups, which indicates that it contains the lowest diversity of bacteria. The Chao index of alpha Diversity indicated that antimicrobial therapy in an HFD scenario substantially decreased the heterogeneity of the gut microbiota (HFD: 296.26 ± 17.66OTU, Abs: 44.66 ± 30.10OTU) (p < 0.001) (**Figure 5B**). After coadministration of AOB, we found that AOB reconstructed the gut microbiota and restored diversity. Principal Coordination Assessment (PCoA) based on the decision UniFrac distance shows a significant difference in the AbsA group’s microbiota composition relative to the Abs group, that decreased the gap compared to that of other three groups (R = 0.7216, P = 0.0010) (**Figure 5C**). The other three groups of PCoA were different. The gut microbiota of the HFD-fed mice after AOB treatment was not the same as that of the HFD group. The group treated with AOB had more widely distributed PCoa coordinates than the HFD group (R = 0.6461, P = 0.0010) (**Figure 5D**). This finding indicates that AOB regulates the structure of the gut microbiota.

### Effects of AOB on Phylum and Genus Level Changes in the Development of Atherosclerosis

**Figure 6:**
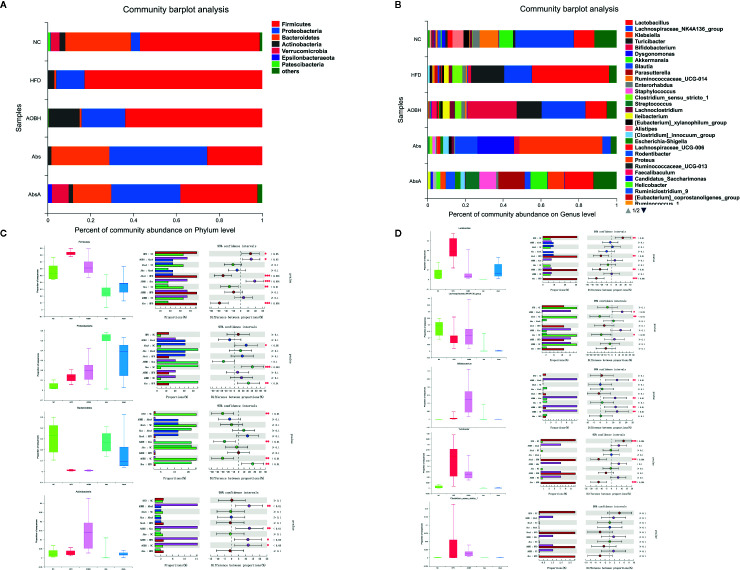
Related phylum- and genus-level bacterial abundance. One-way ANOVA with Tukey-Kramer test was used to assess major group discrepancies, and false discovery rate (FDR) was used to correct multiple tests: *p < 0.05, **p < 0.01, ***p < 0.001. **(A)** Stacked bar plot of taxonomic levels in individual groups. **(B)** One-way phylum level ANOVA bar plot in each group. **(C)** Single-way ANOVA bar plot phylum level Firmicutes, Proteobacteria, Bacteroidetes, and Actinobacteria. **(D)** One-way ANOVA bar plot genus level *Lactobacillus*, Lachnospiraceae NK4A136 group, *Bifidobacterium*, *Turicibacter*, and *Clostridium* *sensu* stricto 1.

At the level of phylum, more than 90% of the microbial populations in each group consisted of Firmicutes, Proteobacteria, Bacteroidetes, and Actinobacteria when compared to the given percentage of the relative abundance (**Figures 6A, C**). Compared with the NC group (55.95 ± 13.15%), the HFD group (82.78 ± 4.13%) exhibited a markedly increased abundance of Firmicutes (p < 0.05), which often enhanced the risk of AS and obesity (Rom et al., 2017; Dong et al., 2019; Ozato et al., 2019). After AOB treatment, the abundance of Firmicutes showed a decreasing trend (63.84 ± 15.10%). No significant change in the abundance of Proteobacteria in the other three groups was observed compared with the two groups treated with antibiotics (Abs: 45.27 ± 21.92%, AbsA: 31.16 ± 21.35%). The Bacteroidetes in the NC group (30.71 ± 15.96%) had the highest abundance compared to the abundance in the HFD group (1.01 ± 0.60%) and the AOBH group (0.89 ± 0.52%). This finding indicates that the intervention of AOB on AS does not take advantage of the change in the abundance of Bacteroidetes. The abundance of Actinobacteria was highest in the AOBH group (14.40 ± 11.40%) (p < 0.05) Compared to other groups, indicating that AOB may have reduced AS by increasing the abundance of Actinobacteria.

At the genomic level, the proportion of microbial abundance of *Lactobacillus*, Lachnospiraceae_NK4A136_group, *Bifidobacterium*, *Turicibacter*, and *Clostridium* *sensu* stricto 1 differed significantly among the NC, HFD, and AOB groups (**Figures 6B, D**). The abundance of *Lactobacillus* was markedly decreased (HFD: 30.98 ± 13.81%, AOBH: 7.71 ± 11.39%) (p < 0.01), and Turicibacte and *Clostridium* *sensu* stricto 1 also showed a decreasing trend after AOB treatment compared to abundance in the HFD group, whereas the abundance of *Bifidobacterium* was markedly increased (HFD: 1.12 ± 1.74%, AOBH: 12.96 ± 10.92%) (p < 0.01); the Lachnospiraceae_NK4A136_group also showed an increasing trend after AOB treatment compared to the NC group.

### The Core Gut Microbiota of Arteriosclerosis Improved by AOB

**Figure 7:**
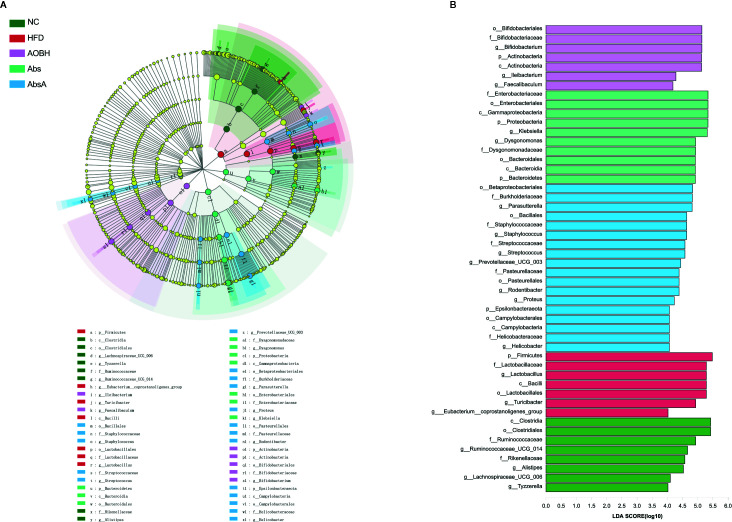
The AOB effect on the composition of gut microbiota. **(A)** Cladogram for taxonomy generated through LEfSe analysis showing prominent shifts in the gut microbiota in each group (score > 4). **(B)** Linear discriminant analysis (LDA) scores (log10) of taxa (score > 4).

**Figure 8:**
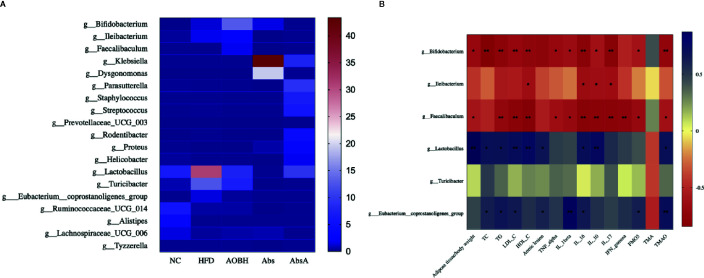
AOB’s effect on key genera and parameters of atherosclerosis. **(A)** Heat map comparison based on the percentages of relative abundance of 19 primary genera across all treatment classes. **(B)** Heat map illustrating the Spearman association between the abundance of main bacterial genera and the parameters of atherosclerosis. *p < 0.05, * bp < 0.01.

We further used LEfSe analysis for highlighting the bacterial phenotypes that led to the variations in the microbiota of gut populations and illustrated 50 gut bacterial clads with major differences (**Figures 7A, B**). In the AOBH group, the phylum actinobacteria (from phylum to genus) was identified to be the abundant one. In addition, the LDA score of the genera *Faecalibaculum* and lleibacterium was also enriched by AOB. In the HFD group, the LDA score of the phylum Firmicutes, class Bacilli, and order *Lactobacillus* (from order to genus) was found to be enriched. Additionally, we examined 19 genera that were considered major genera accountable for the significant differences by LDA score (**Figure 8A**). the AOBH group Compared with the HFD group, was found with less abundance of *Lactobacillus* (HFD: 30.98 ± 13.81%, AOBH: 7.71 ± 11.39%), *Turicibacter* (HFD: 12.52 ± 9.09%, AOBH: 7.21 ± 4.15%), and *Eubacterium*_*coprostanoligenes*_group (HFD: 0.31 ± 0.24%, AOBH: 0.17 ± 0.16%), as well as a higher abundance of *Bifidobacterium* (HFD: 1.12 ± 1.74%, AOBH: 12.96 ± 10.92%), *Ileibacterium* (HFD: 1.75 ± 3.94%, AOBH: 2.44 ± 4.47%), and *Faecalibacterium* (HFD: 0%, AOBH: 0.0012 ± 0.0019%).

### Correlations Between the Atherosclerotic Parameters and Core Gut Microbiota Improved by AOB

We used analysis of Spearman’s correlation (**Figure 8B**) to investigate the associations between some of the atherosclerotic variables and the gut microbiota. The group of HFD, enhanced in *Lactobacillus* and *Eubacterium coprostanoligenes* group shows positive relations with the levels of TC (Lactoba: 0.69, Euba: 0.62), TG (Lactoba: 0.69, Euba: 0.66), LDL-C (Lactoba: 0.72, Euba: 0.63), aortic lesions (Lactoba: 0.66, Euba: 0.59), IL-16 (Lactoba: 0.71, Euba: 0.64), and TMAO (Lactoba: 0.70, Euba: 0.72). *Lactobacillus* was also positively associated with the adipose tissue/body weight ratio (0.87), HDL-C (0.77) and IL-10 (0.76) levels. The *E. coprostanoligenes* group also showed positive relationships with the levels of IL-1-beta (0.86) and FMO3 (0.61). Furthermore, the AOB-induced enhancement in the abundances of *Bifidobacterium* and *Faecalibaculum* showed significant negative relation with the adipose tissue/body weight ratio (Bifidoba: -0.69, Faecaliba: -0.58), TG (Bifidoba: -0.76, Faecaliba: -0.71), LDL-C (Bifidoba: -0.72, Faecaliba: -0.79), HDL-C (Bifidoba: -0.73, Faecaliba: -0.82), TNF-alpha (Bifidoba: -0.69, Faecaliba: -0.64), IL-1 beta (Bifidoba: -0.64, Faecaliba: -0.68), IL-16 (Bifidoba: -0.79, Faecaliba: -0.80), IL-10 (Bifidoba: -0.61, Faecaliba: -0.78), IL-17 (Bifidoba: -0.81, Faecaliba: -0.79), FMO3 (Bifidoba: -0.59, Faecaliba: -0.68), and TMAO (Bifidoba: -0.73, Faecaliba: -0.61) levels. *Bifidobacterium* was also negatively associated with the levels of TC (-0.84), and *Faecalibaculum* showed negative significant correlations with the levels of IFN-gamma (-0.75). Increased abundance of *Ileibacterium* was correlated negatively with HDL-C (-0.63), IL-16 (-0.65), IL-10 (-0.64) and IL-17 (-0.64). We also found that TMA and *Turicibacter* were not significantly correlated with other targets. The applications of these correlations are ambiguous, and to show causality would require further experimentation.

## Discussion

The gut microbiota contains trillions of bacteria. Changes in the microbiota and metabolites derived from it can be used as a signal hub in which environmental influences such as dietary influences can exert effects on the host’s metabolism, response to infection, and immunity (Thaiss et al., 2016). TMAO is produced as a metabolite product as a result of the gut microbiota function and is linked with the pathogenesis of AS. Previous studies show that increased levels of TMAO in blood are positively related with high risks of AS and mortality of all other causes (Wang et al., 2011; Koeth et al., 2013; Schiattarella et al., 2017; Wang et al., 2019). In humans and rodents, gut microbial enzymes convert choline and L-carnitine present in high-fat food into a toxic gas labeled as TMA. TMA travels to the liver through the portal blood circulation where it is oxidized by FMO3 into TMAO (Tang et al., 2013; Shih et al., 2019). When TMAO enters the circulation, it triggers low-grade chronic systemic inflammation. Under constant stimulation of chronic systemic inflammation and high blood lipid levels, the arteries will suffer corresponding damage and produce atherosclerotic lesions (Fatkhullina et al., 2018; Komaroff, 2018; Ridker, 2019). At the population and individual levels, measurements to prevent and reduce AS risk are mainly based on healthy lifestyles, including dietary habits (Piepoli et al., 2016). AOB, as a traditional Chinese medicine prescription provides another option for the prevention of AS and reduction of AS risk.

In the current report, we established that AOB can recover the AS pathological state tempted by HFD in apoE^-/-^ mice. Especially mice lacking apolipoprotein E can easily cause a series of complex vascular lesions symptoms under the stimulation of high-fat diet, which are comparable to human lesions (Emini et al., 2017). In order to determine whether AOB used the gut microbiota to regulate the development of AS, we used antibiotic cocktails to eliminate the effect of gut microbiota in ApoE mice. Antibiotics can eliminate most of the microflora, and it can prove that the drug works by acting on the microbiota of gut (Zarrinpar et al., 2018). Compared to the NC group, the HFD and Abs groups exhibited a series of symptoms of AS, including a high proportion of adipose tissue, high levels of serum lipids. and atherosclerotic lesions. Both high-dose AOB and low-dose AOB administration played a significant role in reducing adipose tissue; serum levels of TG, LDL-C, and TC; and atherosclerotic lesions. Moreover, serum levels, adipose tissue, of TC and atherosclerotic lesions were significantly reduced in the AbsA group compared with the HFD group. AOB has a significant effect on the pathological state of AS, while the gut microbiota and AS are inseparable. After killing the bacteria with antibiotics, we could not improve AS, and the reconstruction of gut microbiota diversity with AOB can play a role in treating AS to a certain extent.

The release of circulating inflammatory cytokines can triggering low-grade chronic systemic inflammation, which will also increase the risk of AS (Komaroff, 2018). Using Magnetic Luminex multicytokines, we demonstrated that AOB can lowering the levels of inflammatory cytokines, including IL-1beta, TNF-alpha, IL-10, IL-17, and IL-6 in serum. Among them, the reduced TNF-alpha, IL-1beta, and IL-17 are active targets for decreasing the progression of CVD (Williams et al., 2019). IL-1 and TNF are central regulators of endothelial cell activation. The activation of NF-kB which in turn upregulates adhesion molecules to promote IL-1 coordinate the recruitment of inflammatory cells to the site of inflammation. This activity especially relevant to atherosclerosis, and in recent plaque imaging studies, IL-17 blockade can prevent the plaque from continuing to expand (Elnabawi et al., 2019). The increase of IL-6 enhance the progression of lipids to damage vessel and IL-10 is associated with the regulation of macrophage activity (Han and Boisvert, 2015; Eltoft et al., 2018). Interestingly, we found that IFN-gamma only has a downward trend in the AOB group, but this trend does not constitute statistical significance. IFN-γ can induce the production of inducible nitric oxide synthase (iNOS) in macrophages, and promote the synthesis of NO. However, IFN cannot stimulate the transcription of related factors such as IL-1 when there is not enough endotoxin stimulation (Sikorski et al., 2011; Lu et al., 2013; Piaszyk et al., 2019; Baidžajevas et al., 2020). Therefore, we speculate that this is due to the relatively short feeding time.

TMAO is produced by foods with choline and L-carnitine and is currently considered to be involved in one of the biological pathways in AS pathogenesis and progression (Abbasi, 2019). Circulating TMAO can release of inflammatory cytokines and induce inflammatory gene expression to damage the artery (Marcus et al., 2016). Using UHPLC**/**MS**/**MS, the contents of TMA and TMAO in serum were determined, and WB was used to measure hepatic FMO3 expression. We demonstrated that AOB can reduce FMO3 expression while lowering the levels of TMAO. With the intervention of antibiotics or atorvastatin, the increase in hepatic FMO3 expression caused by HFD consumption was not controlled, although the corresponding inflammatory cytokine levels were reduced by the intervention of atorvastatin. This indicates that unlike the atorvastatin intervention for AS, AOB can also reduce the oxidation of TMA into TMAO by reducing hepatic FMO3 expression.

The TMAO accumulation process and the pathway through which it facilitates AS includes complex interactions between both the diet and intestinal microbiota (Manor et al., 2018). In the current study, through 16S rRNA high-throughput sequencing, result shows that mice administered antibiotics had significantly lower bacterial diversity than those in the other groups, and the risk of AS in the Abs group of mice was not reduced. This finding shows that the gut involves in the risk of AS, and it is unwise to exclude the impact of the microbiota of gut in the study of AS. Examination of microbiota of gut composition revealed deviations in the intestinal microbiota configuration among the groups. Either at phylum stage a most prevalent microbial species in the host microbiota were Firmicutes, Proteobacteria, Bacteroidetes, and Actinobacteria. Firmicutes and bacteroidetes are considered especially important to public health. Firmicutes can ferment ingested food, while Bacteroidetes uptake and degrade polysaccharides (Xu et al., 2003; Liao et al., 2016). Turnbaugh et al. (2006) found that after transplanting obese germ-free mice with a relative high profusion of Firmicutes and nonobese mouse gut microbiota with a higher relative abundance of Bacteroides, the obese microbiome recipient mice had a higher dietary energy harvesting ability. In addition, Firmicutes was enriched due to HFD intake, and it increases the accumulation of metabolic endotoxins, inflammation, and the risk of AS, while Bacteroidetes does the opposite (Poppleton et al., 2017; Rom et al., 2017; Yoshida et al., 2018; Dong et al., 2019). In this study, we found that the HFD group and the NC group had the highest profusion of Firmicutes and Bacteroidetes, respectively. Compared with the HFD group and NC group, AOB treatment decreased the profusion of Firmicutes and enhanced the profusion of Actinobacteria but did not significantly improve Bacteroidetes. Actinobacteria is considered to have a negative correlation with cholesterol, and changes in Actinobacteria are linked with atherogenic lipid metabolites and proinflammatory cytokines (Li et al., 2017; Nie et al., 2019). Therefore, we believe that AOB inhibits AS by regulating the abundance of Firmicutes and Actinobacteria. Our study also found that Proteobacteria had high abundance in the Abs or AbsA groups, while the differences were not significant in the remaining three groups; therefore, it should have a limited role in the treatment of AS by AOB. At the genus level, compared with no treatment, AOB treatment was found to enhance the profusion of *Bifidobacterium* and reduce the abundance of *Lactobacillus*, which had high abundance in the HFD group. Both *Lactobacillus* and *Bifidobacterium* are well established for their health-related beneficial effects. Furthermore, they can survive in the gastrointestinal transit, possess the potential to adhere to epithelial cells of intestinal and are safe (Marques et al., 2014). However, very few studies have confirmed their efficacy, which is likely due to bias imposed on the designs. A double-blind placebo-controlled study showed that although *Lactobacillus* can reduce cholesterol *in vitro*, it had no effect on volunteers (Lewis and Burmeister, 2005). In addition, studies have shown that even some *Lactobacillus* species, such as *L. ingluviei*, *L. fermentum*, or *L. acidophilus* led to an increase in the body weight, and *L. acidophilus* NCDC 13 did not influence the obesity (Arora et al., 2012), although the combined use of *Bifidobacterium* and *Lactobacillus* and body weight can effectively reduce the weight of mice and reduce cholesterol levels (Bubnov et al., 2017). Our research also suggests that AOB regulates the balance between *Lactobacillus* and *Bifidobacterium* to reduce AS risk.

This is the first study that reports the association between the atherosclerosis-related markers and outcomes of AOB on the gut microbiota composition. Based on LEfSe analysis, our results show that AOB consumption by apoE^−/−^ mice causes a main shift in the microbiota of the gut composition, including increases in the phylum of Actinobacteria (from phylum to genus) and the genera *Faecalibaculum* and lleibacterium as well as decreases in the abundance of the phylum Firmicutes, class Bacilli, and order *Lactobacillus* (from order to genus). Furthermore, based on LDA scores and Spearman’s correlation analysis, we found that *Lactobacillus* and the *Eubacterium*_*coprostanoligenes*_group exhibited positive relationships with atherosclerosis-related markers, while *Bifidobacterium*, Faecalibaculum, and *Ileibacterium* showed significant negative correlations. Our results are in good agreement with previous findings. Gomes et al. (2018) suggested that *Lactobacillus* and *Eubacterium* are related to obesity, which is a risk factor for atherosclerosis. Pallister et al. (2017) found that high levels of *Eubacterium* were linked with enhanced visceral fat mass in people, while *Bifidobacterium* displayed an inverse relationship to *Eubacterium*. In addition, Haro et al. (2016) recently reported that after two years of intervention by dietary, the abundance of *Lactobacillus* and *Eubacterium* genera was lower in the low-fat diet group than in metabolic syndrome patients. Rath et al. (2017), using gene-targeted assays numbering the TMA-producing community and applying them to human fecal samples, found that *Eubacterium* is a potential TMA producer. Interestingly, in our study, AOB resulted in the most obvious changes in the abundance of *Lactobacillus* and *Bifidobacterium* and had a significant correlation with atherosclerosis-related markers. Therefore, we speculate that the therapeutic benefits of AOB on AS are linked with *Lactobacillus* and *Bifidobacterium*, and the regulation of the genus is particularly important. Although the composition mechanism of AOB is not clear enough, it may also contain polyphenols and have similar effects in reducing TMAO (Annunziata et al., 2019a; Annunziata et al., 2019b). Further studies, however, are needed for authenticating the hypothesis.

## Conclusions

Our results propose the noticeable inflection of the gut microbiota by AOB in association with decreased expression of hepatic FMO3 and TMAO concentration *in vivo*. Reduced circulating TMAO prevents the induction of inflammatory cytokine release and reduces low-grade chronic systemic inflammation, ultimately decreasing the risk of atherosclerosis caused by HFD.

## Data Availability Statement

The raw data supporting the conclusions of this article will be made available by the authors, without undue reservation, to any qualified researcher.

## Ethics Statement

The animal study was reviewed and approved by the Institutional Animal Care and Use Committee at Nanjing University of Chinese medicine.

## Author Contributions

Conceived and designed the experiments: BZ, YZ, and HW. Performed the experiments: BZ, YZ, MJ, YW, and JW. Analyzed the data: BZ and YZ. Contributed to the writing of the manuscript: BZ, YZ, WX, WT, and HW. All authors contributed to the article and approved the submitted version.

## Funding

A Project Funded by the Priority Academic Program Development of Jiangsu Higher Education Institutions (Integration of Chinese and Western Medicine).

## Conflict of Interest

The authors declare that the research was conducted in the absence of any commercial or financial relationships that could be construed as a potential conflict of interest.

## Abbreviations

ApoE^−/−^, apolipoprotein E-deficient**;** AOB, *Alisma orientalis* beverage; AOBH, high-dose *Alisma orientalis* beverage; AOBL, low-dose *Alisma orientalis* beverage; Abs, antibiotics cocktail; AbsA, antibiotics cocktail combined with high-dose *Alisma orientalis* beverage; Ato, atorvastatin; AS, atherosclerosis; HFD, high-fat diet; TMA, trimethylamine; TMAO, trimethylamine N-oxide; FMO, flavin−containing monooxygenase; CVD, cardiovascular disease**;** TC, total cholesterol; TG, triglycerides; LDL-C, low-density lipoprotein cholesterol; HDL-C, high-density lipoprotein cholesterol; OTUs, operational taxonomic units; TNF-α, tumor necrosis factor alpha; IL-1β, interleukin-1-beta; IL-16, interleukin-6; IL-10, interleukin-10; IL-17, interleukin-17; PCoA, principal coordinate analysis.
